# Case Report: Lenalidomide as a novel maintenance therapy for metastatic follicular dendritic cell sarcoma

**DOI:** 10.3389/fonc.2026.1801801

**Published:** 2026-05-14

**Authors:** Saarang Kashyap, Esha Sankhala, Jordan Ziegler, Rebecca Godin, Ivan Huang, James Heeter, Spencer Rosant, Kamlesh K. Sankhala

**Affiliations:** 1 Department of Microbiology, Immunology & Molecular Genetics, University of California, Los Angeles, Los Angeles, CA, United States; 2 University of Queensland, Brisbane, QLD, Australia; 3 Precision Next Gen Oncology and Research Center, Los Angeles, CA, United States; 4 Department of Physiological Science, University of California, Los Angeles, Los Angeles, CA, United States; 5 Department of Chemistry and Biochemistry, University of California, Los Angeles, Los Angeles, CA, United States; 6 Department of Biology, University of California, Los Angeles, Los Angeles, CA, United States; 7 Internal Medicine (IM) Hematology Oncology, Cedars Sinai Medical Center, Los Angeles, CA, United States

**Keywords:** follicular dendritic cell sarcoma (FDCS), immunotherapy, lenalidomide, maintenance therapy (MT), metastatic, immunomodulatory

## Abstract

Follicular dendritic cell sarcoma (FDCS) is a rare, low- to intermediate-grade malignancy arising from mesenchymal-derived follicular dendritic cells, which can also present with high-grade pathological features and may rapidly become fatal. Conventional therapeutic strategies, primarily consisting of surgical resection with adjunctive systemic chemotherapy, such as gemcitabine-docetaxel, ifosfamide, doxorubicin, or other regimens, remain inconsistently effective, with median survival notably reduced in advanced or recurrent disease. Given the limited therapeutic options and frequent relapse, there is a critical need for well-tolerated treatments that can improve long-term outcomes for patients with metastatic FDCS. Here, we report the first documented clinical use of the immunomodulatory agent lenalidomide (Revlimid) as maintenance therapy for FDCS following chemotherapy. Remarkably, the patient achieved nearly total sustained remission with exceptional disease stabilization for approximately seven years, experiencing only one manageable recurrence. This clinical response points to lenalidomide as a promising maintenance therapy for metastatic FDCS and may provide further basis for consideration of lenalidomide as a refractory treatment.

## Introduction

FDCS is a mesenchymal dendritic cell neoplasm that arises from follicular dendritic cells, which are critical components of the immune system responsible for presenting antigens to B cells within lymph nodes (1). FDCS accounts for less than 0.4% of all soft tissue sarcomas, and its presentation varies widely depending on the affected site (2). While most cases occur in lymph nodes, extranodal FDCS has been reported in the liver, pancreas, tonsils, and other organs (3). Immunohistochemistry is essential for diagnoses, with CD21, D2-40, CD23, and CD35 recognized as key follicular dendritic cell markers (4, 5), and additional markers such as fascin, somatostatin receptor 2, FDC secreted protein, serglycin, and clusterin further supporting (6, 7). Although these markers aid in identification, diagnosis remains challenging due to the tumor’s rarity, variable morphology, and lack of distinct clinical features.

At present, the primary approach for treating localized FDCS is surgical resection, representing approximately 85% of all cases, but there is no defined standard of care for metastatic FDCS (7, 8). However, FDCS recurrence remains common, with pooled analysis showing local recurrence in 28.1% and distant metastasis in 27.2% of patients following initial treatment (9). For advanced FDCS, various chemotherapy regimens, such as CHOP (cyclophosphamide, doxorubicin, vincristine, and prednisone), gemcitabine, and ifosfamide amongst others have been employed, yet they generally offer only transient disease control and limited success in remission (6). While some data suggests that radiation therapy (RT) may reduce locoregional recurrence rates, this does not consistently translate into improved disease outcomes (10). For example, in head and neck FDCS, patients receiving surgery plus RT had lower locoregional recurrence rates (15% for both vs. 45% for surgery) compared to surgery alone; however, there was no significant difference in disease free survival, time to first recurrence, death rate, and 5 year overall survival (11).

Here we present the first documented case of using lenalidomide as a maintenance therapy in metastatic FDCS. Lenalidomide is an immunomodulatory drug widely used in the treatment of multiple myeloma and certain lymphomas (12, 13). It has several mechanisms of action, including promoting T cell activation, enhancing natural killer (NK) cell activity, and inhibiting angiogenesis (14). This patient’s sustained response to lenalidomide, with only a single recurrence over a nearly 7 year timespan, suggests that it offers therapeutic benefit as a maintenance strategy in FDCS, particularly when conventional treatments fail to prevent relapse.

## Case presentation

**Figure 1:**
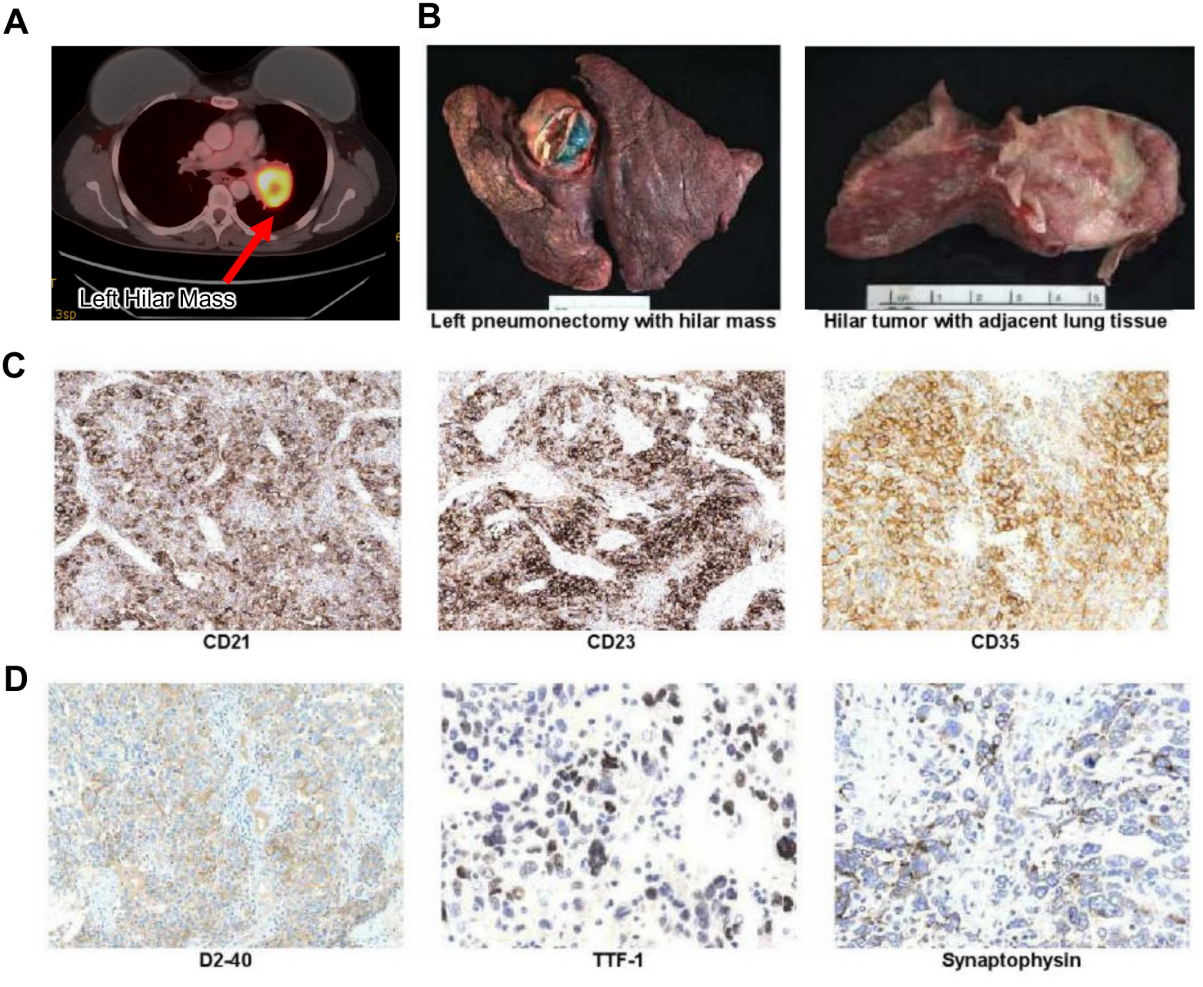
**(A)** PET scan showing initial diagnosis of FDCS as a hypermetabolic left hilar mass. **(B)** Gross image of left pneumonectomy specimen demonstrating a 4.9 cm heterogeneous hilar mass inseparable from the left upper lobe and mediastinum **(C)** Immunohistochemistry (IHC) shows tumor cell positivity for FDCS markers CD21, CD23, and CD35. **(D)** Immunohistochemistry shows focal positivity of D2-40, TTF-1 and synaptophysin.

The patient is a 54-year-old female with past medical history including right breast ductal carcinoma *in situ*, for which she underwent a right mastectomy and prophylactic left mastectomy with bilateral breast reconstruction in 2011. She was initially referred in June 2015 after a chest X-ray revealed a mass in the left perihilar region. CT and PET scans confirmed a 5 cm, intensely PET-avid left hilar mass, with no evidence of distant disease (**Figure 1A**). The patient underwent fiberoptic bronchoscopy and cervical mediastinoscopy for staging of lung cancer, during which a distal left mainstem lesion was biopsied (negative for malignant cells) and multiple mediastinal lymph nodes were sampled, which were anthracotic and negative on frozen section. Shortly after, the patient underwent a left pneumonectomy in which a large tumor was resected, with clear surgical margins and no involvement of hilar lymph nodes (**Figure 1B**), and final pathology identified her tumor as a high-grade FDCS.

**Figure 2:**
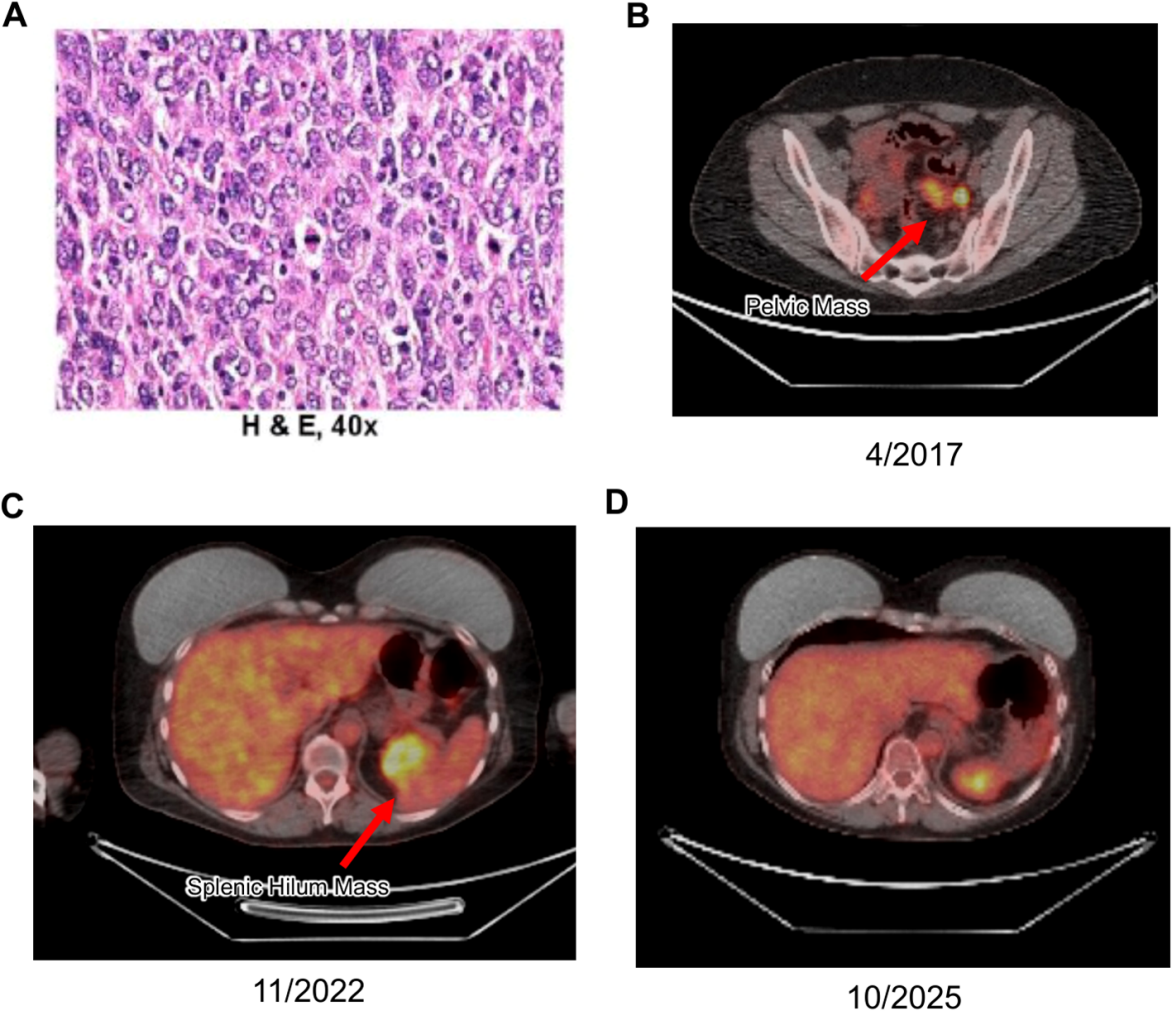
**(A)** Hematoxylin and eosin (H&E) staining at 40x magnification reveals epithelioid and spindle cells with marked cytologic atypia and areas of necrosis. **(B)** First recurrence of FDCS identified by PET scan, demonstrating a new hypermetabolic metastatic lesion in the left pelvis a year after patient received 2 cycles CHOP chemotherapy. **(C)** PET scan capturing subsequent recurrence as an intensely active, heterogeneous mass at the splenic hilum after nearly 4 years on lenalidomide. **(D)** Follow-up PET imaging in October 2025 showing no evidence of recurrence or active disease.

The tumor cells demonstrated strong immunohistochemical positivity for CD21, CD23, and CD35 and were negative for keratin and other lineage-specific markers (**Figure 1C**). Focal expression of D2–40 was noted, providing further support for a diagnosis of FDCS (15, 16). Aberrant focal expression of TTF-1 was noted (**Figure 1D**); however, a lung or thyroid primary tumor origin was ruled out considering negativity for markers Napsin A and PAX-8. Notably, TTF-1 expression has been reported in FDCS of the head and neck region (17, 18). The tumor also demonstrated focal aberrant expression of synaptophysin (**Figure 1D**). Although synaptophysin is a neuroendocrine marker not typically associated with FDCS, there are reports of weak focal positivity expression of synaptophysin in FDCS (19). This finding may reflect aberrant differentiation and further underscores the tumor’s high-grade, pleomorphic nature. The presence of high-grade features, such as significant cytologic atypia, extensive necrosis, and a high proliferative index indicated a potentially aggressive clinical course (**Figure 2A**). After consultation with sarcoma experts. After extensive conversation, adjuvant CHOP chemotherapy was initiated after the left pneumonectomy.

However, following her initial cycle of CHOP chemotherapy in early 2016, the patient developed severe side effects, including significant nausea, vomiting, and bone pain. Despite adjustments to antiemetic therapy, she decided to discontinue CHOP after two cycles due to persistent side effects. Follow-up CT scans showed no further evidence of disease progression, and she was transitioned to regular surveillance. Patient experienced recurrence in left ovary, and a left salpingo-oophorectomy was performed in November 2016, with pathology revealing a high-grade malignant neoplasm consistent with follicular dendritic cell sarcoma involving the ovarian parenchyma and surface with paraovarian extension, and similar high-grade tumor identified in tissue adjacent to the sigmoid colon.

In 2017, the patient experienced abdominal pain and bloating. An April PET scan identified a 2.2 cm pelvic mass, and biopsy confirmed FDCS (**Figure 2B**). In May of the same year, she underwent extensive tumor debulking with resection of all identified macroscopic disease, including rectosigmoid resection, hysterectomy with right salpingo-oophorectomy, and excision of multiple peritoneal and pelvic nodules. The final pathology demonstrated no residual tumor at resection margins and no visible gross residual disease was left behind at the conclusion of the operation.

Intraoperatively, multiple tumor nodules were found in the left ovary, fallopian tube, rectal serosa, bladder wall, and sigmoid mesentery. Pathology confirmed metastatic FDCS, and clear margins were achieved in most resected tissues, though the disease was extensive. Postoperatively, the patient received six cycles of paclitaxel and gemcitabine chemotherapy, achieving initial disease stabilization. In November 2018, due to failure of previous chemotherapy regimens, the patient was started on lenalidomide, 4 months after paclitaxel and gemcitabine treatment. Patient experienced significant nausea and vomiting after first lenalidomide 20mg dose and held further doses. Patient restarted lenalidomide at reduced 5mg once daily continuous dosing the following month. Patient reported no side effects at this dosing level at 4-week follow up and increased to 10mg once daily continuously. This rechallenge at the higher dose was well-tolerated without significant side effects or lab toxicities at subsequent 3-month follow-ups. Throughout the course of treatment with lenalidomide 10mg once daily, patient denied side effects and did not require anti-emetics, other supportive medications, or dose reductions.

By 2019, imaging indicated stable disease with no new metastases. A PET scan at the end of the year showed minimal residual metabolic activity. The patient continued on 10 mg lenalidomide daily, showing no indication of new metastases through 2020, 2021, and 2022, as confirmed by regular CT and PET scans. In late 2022, a PET scan revealed a moderately to intensely active heterogeneous mass in the splenic hilum, which appeared larger and more active compared to prior imaging, raising strong suspicion of a neoplastic lesion (**Figure 2C**). In January 2023, the patient underwent surgery, which included the excision of the recurrent tumor, an in-continuity distal pancreatectomy and splenectomy, and a partial omentectomy. Pathology confirmed the mass as metastatic FDCS. Following recovery, the patient resumed lenalidomide at 10 mg once daily continuously, with ongoing regular monitoring showing stable disease and no new activity.

**Figure 3:**
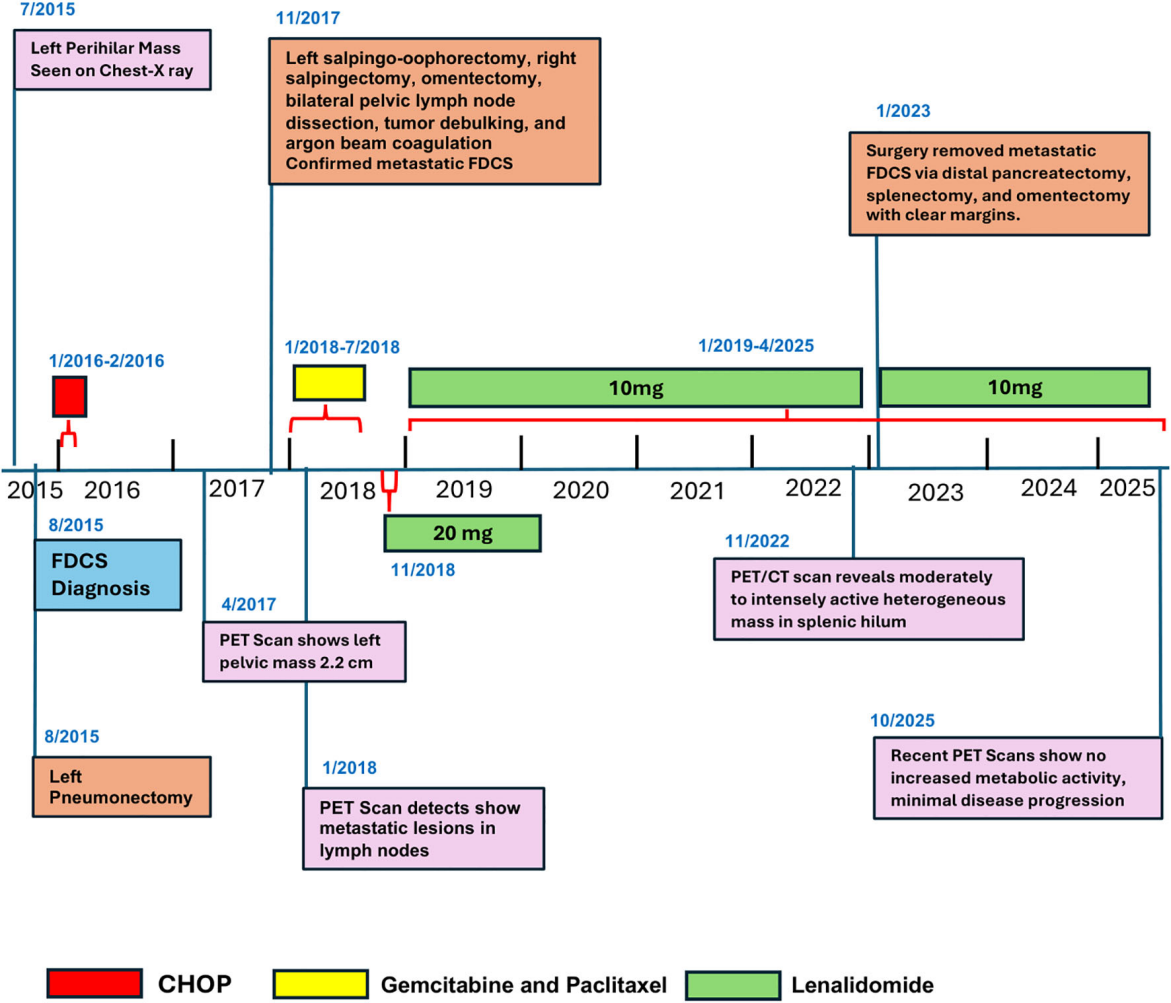
Timeline illustrating the patient’s clinical course from initial FDCS diagnosis through sequential recurrences, surgical interventions, systemic treatments, and current disease-free status.

Through late 2023 and 2024, PET/CT (skull to mid-thigh) scans showed no new areas of increased metabolic activity, and there was no reported disease relapse (**Figure 2D**). A chronological overview of the patient’s clinical course, including diagnosis, recurrences, surgical procedures, systemic therapies, and the achievement of disease-free status is given in **Figure 3**. Continuous monitoring with Natera’s circulating tumor DNA (ctDNA) testing shows stable ctDNA levels, with no notable increases since early 2023 (**Supplementary Figure 1**).

Throughout her follow-up visits, extending to late October 2025, the patient has generally reported a good quality of life, managing mild hot flashes and occasional bone pain unrelated to lenalidomide with over-the-counter medications. The patient continues to have PET/CT (skull to mid-thigh) scans every 6 months with no further evidence of metabolically active recurrent or metastatic disease in the chest, abdomen, or pelvis, or other regions. She remains physically active, able to exercise regularly without major limitations, and is overall satisfied with her treatment on lenalidomide.

## Discussion and conclusion

Follicular dendritic cell sarcoma (FDCS) is an extremely rare malignancy arising from follicular dendritic cells within the germinal centers of lymphoid tissue. In the revised fourth edition of the World Health Organization (WHO) classification, FDCS was categorized among histiocytic and dendritic cell neoplasms; however, in the most recent fifth edition it has been reclassified under mesenchymal dendritic cell neoplasms within the broader category of stroma-derived neoplasms of lymphoid origin (20). This updated classification reflects growing evidence that FDCS demonstrates mesenchymal features and may behave biologically more similarly to sarcomas than to classical lymphoid malignancies (21). Correspondingly, previous reports have suggested that sarcoma-based chemotherapy regimens, particularly gemcitabine- and taxane-containing combinations, may produce meaningful responses in advanced disease (22, 23). At the same time, it is important to recognize that favorable long-term outcomes in individual cases may also be influenced by the extent of surgical resection. In our patient, the achievement of gross complete resection during tumor debulking likely contributed to disease control and may represent a confounding factor when interpreting the durability of response observed with subsequent maintenance therapy. However, as seen in many patients with advanced disease, durable long-term control with cytotoxic therapy alone remains difficult to achieve, highlighting the need for additional therapeutic strategies to prevent recurrence and maintain disease control.

There has been limited exploration into the role of maintenance therapy in FDCS. A recent report described two patients with bulky abdominopelvic FDCS who were treated with a chemoimmunotherapy regimen combining gemcitabine and docetaxel with pembrolizumab (24). One patient, who achieved a complete metabolic response after six cycles, continued on the same regimen as maintenance therapy. The other patient, who had hepatic metastases, transitioned to pembrolizumab (PD-1 inhibitor) maintenance following initial therapy and demonstrated tumor shrinkage and symptom improvement. Both patients exhibited intermediate PD-L1 expression and sustained clinical responses with good tolerability. In another instance, the use of pembrolizumab led to complete remission and a progression-free survival of 24 months in a patient with metastatic tonsillar FDCS following chemoradiotherapy (25). These cases suggest that immunomodulatory treatment has been successful when applied towards FDCS.

Considering previous immunotherapy approaches in preventing FDCS spread and recurrence, lenalidomide represents a compelling candidate given its immunomodulatory properties and previously established role in hematologic malignancies (26). Lenalidomide has become a cornerstone of maintenance therapy in newly diagnosed multiple myeloma (NDMM) following autologous hematopoietic stem cell transplantation (auto-HSCT) (27, 28). Multiple randomized clinical trials, like CALGB 100104, IFM 2005-02, and GIMEMA RV-MM-PI-209, have demonstrated significant progression free survival with hazard ratios of 0.38 and 0.53 in the CALGB and IFM trials, respectively, compared to placebo or no maintenance therapy (29). A comprehensive patient-level meta-analysis of three randomized controlled trials demonstrated that lenalidomide maintenance therapy following auto-HSCT in newly diagnosed multiple myeloma not only significantly prolonged progression-free survival but also conferred a clinically meaningful overall survival benefit, reducing the risk of death by 25% compared to placebo or observation, with a favorable trend also observed in earlier analyses (30).

While these data implicate lenalidomide as an important maintenance therapy for multiple myeloma, the mechanistic rationale for using lenalidomide in FDCS is supported by parallels with follicular lymphoma (FL). Lenalidomide, particularly when combined with rituximab, has significantly improved progression-free survival (PFS) in relapsed or refractory FL, even in rituximab-refractory disease (31). In the RELEVANCE trial, a combined regimen of rituximab and lenalidomide demonstrated comparable efficacy to rituximab plus chemotherapy in previously untreated FL, with a 3-year PFS rate of 77% and fewer grade 3–4 hematologic toxicities such as neutropenia (32). Given the overlapping immunophenotypic features between FDCS and FL, both of which can present as nodular proliferations with spindle or atypical large cells and may express dendritic or lymphoid markers such as CD21, CD35, and S-100, therapies effective in FL may reasonably be considered in FDCS, especially in relapsed or refractory settings (33–35).

In conclusion, using lenalidomide may be a sound and well-tolerated therapeutic option for patients with metastatic FDCS, especially when standard chemotherapy is inadequate or contraindicated. In the case presented here, the use of lenalidomide led to long-term disease stabilization extending approximately 7 years with one recurrence, suggesting that this immunomodulatory therapy may offer durable control in FDCS. As conventional treatment options remain limited and long-term toxicities of chemotherapy are a concern, the introduction of lenalidomide as a maintenance therapy may represent a paradigm shift in the management of FDCS. Importantly, this case provides proof-of-concept that immunomodulatory maintenance therapy can meaningfully alter the progress of metastatic FDCS, supporting a new strategy for long-term disease control in a malignancy with few durable options.

## Methods

### Case report

This case report presents a patient diagnosed and treated for follicular dendritic cell sarcoma (FDCS) at Cedars-Sinai Medical Center, Los Angeles, CA. The patient provided written informed consent for use of anonymized clinical data and imaging for publication. Clinical data were retrospectively collected through comprehensive chart review and included pathology, surgical history, imaging, treatment regimens, laboratory results, and follow-up outcomes. Quality of life assessment was conducted through the physician’s clinical judgement.

### Histopathology and immunohistochemistry

Diagnostic confirmation of FDCS was performed through histopathological evaluation and immunohistochemistry (IHC). Surgical specimens, including pneumonectomy and metastatic tissues, were formalin-fixed and paraffin-embedded (FFPE). Tissue sections (approximately 4 µm thick) were stained with hematoxylin and eosin (H&E) for morphological evaluation. Immunohistochemical staining was conducted using automated platforms at Cedars-Sinai Medical Center, with standard antigen retrieval protocols utilizing heat-induced epitope retrieval (HIER) techniques. The presence of FDCS was verified by diffuse positive staining for follicular dendritic markers (CD21, CD23, CD35), focal positivity for TTF-1 and D2-40, and absence of staining for epithelial (pancytokeratin) and lineage-specific markers (CD3, CD20, CD45, Desmin, S100, Napsin A, PAX-8, among others). Appropriate positive and negative controls were included to ensure the accuracy of staining results.

### Imaging and disease monitoring

Longitudinal disease monitoring was conducted via serial 18F-FDG PET/CT and CT scans from 2015 through 2025. Imaging data was collected and reviewed by board-certified radiologists at Cedars-Sinai Medical Center. Imaging studies assessed both metabolic activity and anatomical changes across thoracic, abdominal, and pelvic regions. Circulating tumor DNA (ctDNA) testing (Signatera™, Natera Inc.) was incorporated as a non-invasive method to track minimal residual disease and treatment response from 2023 onward.

## Data Availability

The original contributions presented in the study are included in the article/**Supplementary Material**. Further inquiries can be directed to the corresponding author.
