# Supplementary material for: Case Report: Lenalidomide as a novel maintenance therapy for metastatic follicular dendritic cell sarcoma
Source: Front Oncol. 2026 May 14;16:1801801. doi: 10.3389/fonc.2026.1801801 (PMC13215918; doi:10.3389/fonc.2026.1801801)
Supplement: Supplementary file 2 [file DataSheet2.pdf]

Supplementary Figures

FINAL RESULTS SUMMARY

Signatera Negative

MTM/mL: Not Detected

Mean tumor molecules per mL is calculated based on the mean of ctDNA molecules detected per mL of the patient's plasma. See Limitations section below.

Historical Results

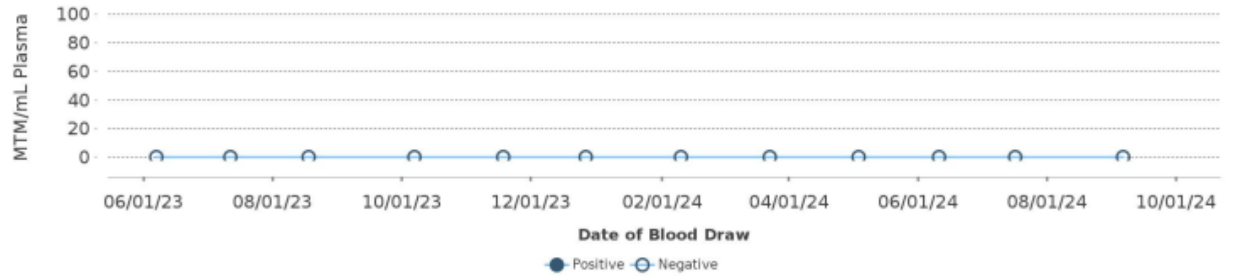

Date	Reported MTM/mL
Sep 06, 2024	0.00
Jul 17, 2024	0.00
Jun 11, 2024	0.00
May 04, 2024	0.00
Mar 23, 2024	0.00
Feb 10, 2024	0.00
Dec 27, 2023	0.00
Nov 18, 2023	0.00
Oct 07, 2023	0.00
Aug 18, 2023	0.00
Jul 12, 2023	0.00
Jun 07, 2023	0.00
Feb 17, 2023	0.00

Supplemental Figure 1. Circulating Tumor DNA testing shows no significant MTM/mL Plasma after recurrence of FDCS
